# Water uptake by gecko β-keratin and the influence of relative humidity on its mechanical and volumetric properties

**DOI:** 10.1098/rsif.2022.0372

**Published:** 2022-09-21

**Authors:** Marzieh Khani, Tobias Materzok, Hossein Eslami, Stanislav Gorb, Florian Müller-Plathe

**Affiliations:** ^1^ Eduard-Zintl-Institut für Anorganische und Physikalische Chemie, Technische Universität Darmstadt, Alarich-Weiss-Str. 8, 64287 Darmstadt, Germany; ^2^ Department of Chemistry, College of Sciences, Persian Gulf University, Boushehr 75168, Iran; ^3^ Zoological Institute, Functional Morphology and Biomechanics, Kiel University, Am Botanischen Garten 1-9, 24118 Kiel, Germany

**Keywords:** wet adhesion, gecko adhesion, water solubility in keratin, mechanical properties of gecko keratin

## Abstract

Grand canonical ensemble molecular dynamics simulations are done to calculate the water content of gecko β-keratin as a function of relative humidity (RH). For comparison, we experimentally measured the water uptake of scales of the skin of cobra *Naja nigricollis*. The calculated sigmoidal sorption isotherm is in good agreement with experiment. To examine the softening effect of water on gecko keratin, we have calculated the mechanical properties of dry and wet keratin samples, and we have established relations between the mechanical properties and the RH. We found that a higher RH causes a decrease in the Young's modulus, the yield stress, the yield strain, the stress at failure and an increase in the strain at failure of the gecko keratin. At low RHs (less than 80%), the change in the mechanical properties is small, with most of the changes occurring at higher RHs. The changes in the macroscopic properties of the keratin are explained by the action of sorbed water on the molecular scale. It causes keratin to swell, thereby increasing the distances between amino acids. This has a weakening effect on amino acid interactions and softens the keratin material. The effect is more pronounced at higher RHs.

## 1. Introduction

The gecko toe consists of millions of setae branched up into bundles of finer sticky spatulae, which can follow almost any surface topography [1]. A single seta of the Tokay gecko *Gekko gecko* generates an adhesion force of 200 µN and that of a single spatula exceeds 20 nN [2]. The resulting calculated adhesion force is more than 100 times larger than the gecko body weight [3]. Numerous studies have been done to understand the mechanism of gecko adhesion force. Experimental works by Autumn *et al*. [4,5] have addressed to the role of van der Waals interactions in the gecko adhesion. Atomic force microscopy experiments by Huber *et al*. [1,6] have shown that increasing humidity significantly enhances the gecko adhesion, and they concluded that capillary forces can also be regarded as a mechanism of gecko adhesion, if water is present. However, there are competing explanations for better adhesion at higher relative humidity (RH). Hypotheses include the modification of short-range interactions due to water adsorption by the spatula [1], the influence of water on the surface chemistry of the contacting surface [7], capillary forces [6,8] and acid–base interactions [9] between polar lipid head groups and hydrophilic surfaces. Puthoff *et al*. [10] and Prowse *et al*. [11], however, reported that the humidity-increased gecko adhesion is due to humidity-induced seta softening, which causes a better contact of the setae to rough surfaces.

The main component of the gecko setae is β-keratin [12]. Due to its high tensile strength and high Young's modulus (2 GPa) [13], the seta has the ability to follow to surface irregularities and to deform before failure. Prowse *et al*. [11] found that an increase in RH reduces the stiffness and increases the strain to failure of the gecko keratin, supporting the hypothesis that water has a plasticizing (softening) effect.

Bioinspired materials have been thought to be a good model to study the mechanism of wet adhesion in gecko [14,15]. In this respect the main focus has been on the geometrical design of microfibrillar patterns, tuning the geometry and softness of tips. No clear conclusion about competing effects related to different RHs on adhesion can be drawn from these reports. The adhesion of synthetic polydimethylsiloxane hydrophobic microfibrils to polar surfaces is either unaffected [16] by RH or it decreases [17] at high RHs. On the other hand, nanopillars made of diblock copolymers containing a more hydrophilic component, poly(2-vinylpyridine), show a significant increase in the adhesive strength with increasing RH [18]. The result of a recent study [19], however, shows that adhesion increases with increasing RH for both hydrophobic and hydrophilic gecko-inspired synthetic nanopillars, which was attributed to the softening of the nanopillars by water.

In addition to experiments, molecular simulations have been employed to obtain microscopic information on the gecko adhesion. A review by Sauer [20] discusses computational modelling schemes of gecko adhesion at different length scales. Very recently, we have developed coarse-grained [21,22] and united atom [23] models of gecko keratin to study the mechanism of wet and dry adhesion. We found that both capillary bridges and individual water molecules facilitating the keratin–surface contacts made wet keratin more adhesive than dry keratin.

In this work, we perform united atom [23] molecular dynamics simulations to calculate several macroscopic properties of gecko keratin, including the amount of swelling, Young's modulus and Poisson's ratio, as a function of the water content of the keratin. The first purpose is to establish the relation between RH and the equilibrium water content of gecko keratin by grand canonical ensemble (GCE) molecular dynamics simulations. For comparison, we have measured experimentally the water uptake of dorsal and ventral scales (β-keratin) of the snake *Naja nigricollis* as a function of RH. The material is thought to be chemically similar to gecko setae and available in larger quantities. Experimental or computational reports on the solubility of water in β-keratin and properties following from it are scarce. This is in marked contrast to α-keratin, where the cosmetics and textile industries ensure a continuous flux of data [24–26]. Secondly, we want to know how macroscopic mechanical properties of gecko keratin change with water content and, thereby, the ambient RH. This also serves our third objective, namely to elucidate further the molecular mechanism responsible for the stronger adhesion of the gecko keratin to surfaces at higher humidity. As mentioned above, while some reports credit the softening of keratin by water, the results of our recent simulations indicate individual water molecules acting as contact facilitators [23]. To discriminate between the two mechanisms, we calculate the elastic properties of keratin as a function of RH. If the softening mechanism were to dominate the humidity-enhanced wet adhesion of gecko keratin, it would necessitate a marked decrease of the elastic constants at high RHs. Using the calculated solubilities of water, we establish a connection between elastic properties of gecko keratin and RH.

## 2. Theory

There are a number of phenomenological models for investigation of the sorption isotherms of small penetrants in polymers. A modified version of the Brunauer, Emmett and Teller [27] model, the so-called Guggenheim–Anderson–de Boer (GAB) model [28], has been found to successfully fit experimental type II isotherms (also called sigmoidal isotherms) up to high partial pressures. Although it was originally proposed for the adsorption of gases on solid substrates, where gas condensation occurs at higher pressures than in synthetic or biological polymers, the GAB equation has been successfully employed to fit experimental water solubilities in α-keratin [29]. The GAB isotherm, which is based on a multilayer adsorption model, reads as

2.1 $$C(a)=C_{m}\frac{bKa}{\left(1-ba)(1-ba+bKa\right)},$$

where *C*(*a*) is the sorbate content in equilibrium with a sorbate activity *a* in the vapour phase, *C*_m_ is the sorbate content corresponding to an adsorbed monolayer, and *b* and *K* are two constants. In the case of water, *a* is expressed in terms of RH. The parameters *C* and *C*_m_ are expressed in terms of weight percent on dry basis (grams of sorbate per 100 g of sorbent). The dimensionless parameters *K* and *b* are related to the differences between the bulk heat of liquefaction of vapour and the heats of adsorption on the first layer (for *K*) and to the rest of the layers (for *b*) as [28,29]

2.2 $$K=e^{\Delta H/RT},$$

where Δ*H* is the difference between the heat of liquefaction and the heat of adsorption of sorbate, *R* is the universal gas constant and *T* is the temperature. In spite of the concept of adsorption of different layers being slightly out of place for absorption into a material, the GAB isotherm successfully fits water sorption data in α-keratin [29]. It, however, contains three unknown parameters, to be adjusted against experimental data.

We intend to improve the molecular understanding of the process of water sorption in gecko keratin. This necessitates a model, in which the detailed chemical structures of both keratin and water are taken into account. The sorption isotherm of water in gecko keratin is calculated by molecular dynamics simulations in the GCE [30,31]. Thermodynamically, the equilibrium between water molecules in the vapour phase and sorbed in the keratin is reached at constant temperature, *T*, and constant pressure, *P*, and equal chemical potentials of water in both phases. The following equation between the excess chemical potentials of water in the gecko keratin and vapour applies:

2.3 $$\mu_{w,k}^{\mathrm{ex}}(T,P)=\mu_{w,g}^{\mathrm{ex}}(T,P)-k_{B}T\ln\left(\frac{C\rho_{k}}{100\rho_{w,g}}\right),$$

where $\mu_{w,k}^{\mathrm{ex}}$ and $\mu_{w,g}^{\mathrm{ex}}$ are the excess chemical potentials of water in the gecko keratin and the gas, respectively, *k*_B_ is Boltzmann's constant, *ρ*_w,g_ is the mass density of water in the gaseous phase and *ρ*_k_ is the mass density of the gecko keratin in the water–keratin mixture, and the factor 1/100 ensures proper unit conversion for the density of water in the keratin phase to grams of water per 100 g of keratin. The excess chemical potential, *μ*^ex^, is defined as the difference between the actual chemical potential of a species and that of its ideal gas at the same density and temperature. The chemical potential of the ideal gas is [32]

2.4 $$\mu^{\mathrm{id}}=k_{B}T\ln\left[{\left(\frac{h^{2}}{2\pi mk_{B}T}\right)}^{3/2}\frac{N}{V}\right]+B(T),$$

where *N* is the number of molecules, *V* is the volume, *m* is the molecular mass, *h* is Planck's constant and *B*(*T*) stands for the contribution of internal degrees of freedom of the sorbate to the chemical potential of the ideal gas. The logarithmic term on the right-hand side of equation (2.3) is the difference between the ideal gas contributions to the chemical potentials of water in gaseous and keratin phases.

In the GCE the temperature, the volume, and the chemical potential are set as independent thermodynamic parameters and water molecules are exchanged between the gecko keratin phase and an ideal gas reservoir. In equilibrium, the number of water molecules in the gecko keratin phase fluctuates around an average value, consistent with initially fixed conditions, *μVT*.

## 3. Model and simulation details

Our united-atom gecko keratin model uses the GROMOS 54A7 force field [33]. The gecko β-keratin protein contains a β-sheet region, which associates into dimers and further polymerizes into fibrils [12]. The β-sheet is surrounded by intrinsically disordered protein (IDP) domains [23]. Water can infiltrate the loose IDPs better than dense, structured proteins [34]. Furthermore, since the fibrils are on the inside, only the IDP domains are considered to be available for adhesive contacts [23]. Therefore, we consider only the amorphous regions of the protein and omit the β-sheet nanofibrils, i.e. the amorphous sections of β-keratin are used to model the gecko spatula keratin. The simulated β-keratin sample contains 18 protein molecules, each consisting of 96 amino acids. Every molecule contains 22 cysteine amino acids, which may form intermolecular disulfide bonds, leading to a cross-linked elastomeric network. In this sample, 33% of cysteine amino acids (7.5% of total amino acids) are cross-linked through disulfide bridges. Thus, the average peptide strand between two disulfide bridges is 13 amino acids. More details of the β-keratin model are given in our previous publication [23].

For water, the extended simple point charge, SPC/E, model [35] is used. The water molecule was kept rigid using the SHAKE algorithm [36]. The parameters for unlike Lennard-Jones interactions were determined using Lorentz–Berthelot mixing rules. All molecular dynamics simulations were done using our simulation package YASP [37]. The equations of motion were integrated using the velocity-Verlet algorithm, and a Nosé thermostat [38] was used to keep the temperature at 300 K. The time step was 1.5 fs. An atomic Verlet neighbour list was used, which was updated every 15 time steps. The neighbours were included in the list if they were closer than 1.1 nm and the cutoff for nonbonded interactions was 1.0 nm. A reaction-field correction [37] was used for the electrostatic interactions. The effective dielectric constant was defined as the average dielectric constants of water and keratin, i.e the linear addition of the volume fraction of each component multiplied by its corresponding dielectric constant. The reaction field dielectric of keratin was taken equal to the experimental value, ≈ 4 [39], and that of water was taken equal to 72 [40]. The calculated effective dielectric constants for water–keratin mixtures are in good agreement with experiment [39]. For example, the calculated dielectric constants at 5, 10 and 17.2 g water per 100 g keratin are 5.8, 8.8 and 12.7, respectively, which are close to experimental values (6, 8.6 and 17, respectively).

To perform GCE molecular dynamics simulations, we have prepared initially relaxed configurations of gecko keratin mixed with water (see §4.1) in the *NPT* ensemble. The details of the GCE simulation method are described elsewhere [30]. Here, we just note that our approach is based on the extended Hamiltonian formalism. The kinetic and potential energy terms for an additional degree of freedom, *λ,* which determines the coupling of the system to the water reservoir, are included in the Hamiltonian, and its equations of motion are solved. The system is composed of real particles (keratin plus water molecules) and one fractional water molecule, whose potential energy of interaction with the rest of the particles is scaled by the coupling parameter, 0 < *λ* < 1. The governing equation for the motion of the coupling parameter is [30,31]

3.1 $$W\frac{d^{2}\lambda }{dt^{2}}=-\frac{\partial U(\lambda )}{\partial \lambda }+\mu_{w,k}^{\mathrm{ex}},$$

where *W* is the mass associated with the additional degree of freedom, *λ*, and *U*(*λ*) is the potential energy of interaction between the fractional water molecule and the other particles in the system. Governed by equation (3.1), the coupling parameter varies dynamically. When it reaches 0, the fractional molecule is removed from the system and another water is designated the fractional molecule. When *λ* reaches 1, the fractional molecule is converted into a regular fully interacting molecule and a new fractional molecule is added to the system. At equilibrium, the density of water sorbed in the keratin fluctuates around an average value, consistent with the target values of temperature, volume and the excess chemical potential for water in the gecko keratin phase. We used a nonlinear coupling scheme, proposed previously [30], to make the potential energy of interaction of the fractional particle a continuous, slowly varying function of *λ*. The values of the inertial parameters (masses) for temperature and coupling parameter were both 5.0 kJ mol^−1^ ps^2^. A standard tail correction was used for the potential energy of the fractional water molecule [31].

To relate the water chemical potential to the RH, the chemical potential of water in the vapour phase is needed as well. We do a second GCE simulation of the gas phase at the temperature of the gecko keratin phase, and adjust the target chemical potential of the vapour phase according to the pressure expansion of the excess chemical potential in the keratin phase, i.e. [30,31]

3.2 $$\mu_{w,k}^{\mathrm{ex}}(T,P)\approx \mu_{w,k}^{\mathrm{ex}}(T,P^{\prime })+v_{w,k}(P-P^{\prime })+k_{B}T\ln\left(\frac{P^{\prime }}{P}\right),$$

where *v*_w,k_(*T*, *P*) is the molar volume of water in the gecko keratin phase, *P*′ is the pressure of the gecko keratin phase (*P*′ is the average pressure over the time window for which the density of water, in the GCE simulation of the keratin phase, fluctuates around its average) and *P* is the equilibrium vapour pressure (unknown), calculated from the GCE simulation of the gas phase. Previously we have employed this method for the calculation of sorption isotherms of small molecules in polymers [31,41] and biomembranes [42] and phase equilibria in Janus colloidal particles [43–45].

In this work to generate initial water–keratin mixtures, we set a relatively high target chemical potential to add water molecules to the keratin sample. Then we perform *NPT* ensemble simulations (50 ns) to generate relaxed water–keratin mixtures at specified water concentrations. The GCE simulations always start from initially relaxed configurations. The molar volume of water in the gecko keratin phase, *v*_w,k_(*T*, *P*), to be used in equation (3.2) is obtained from the results of *NPT* ensemble simulations. For each phase, relatively long GCE simulations (30 ns) are done to obtain the average density of water.

For calculation of mechanical properties, we start from relaxed configurations, generated from the GCE simulations. The simulation box is uniformly deformed along one axis at a constant rate (0.00001 nm ps^−1^) and a semi-isotropic Berensen barostat [35] is employed to keep the pressure constant at 101.3 kPa. The simulation box remains incompressible along the deformation axis, i.e. the size of the simulation box fluctuates in the perpendicular (to the deformation axis) directions.

## 4. Results and discussion

### 4.1. Water solubility in gecko keratin

**Figure 1.**
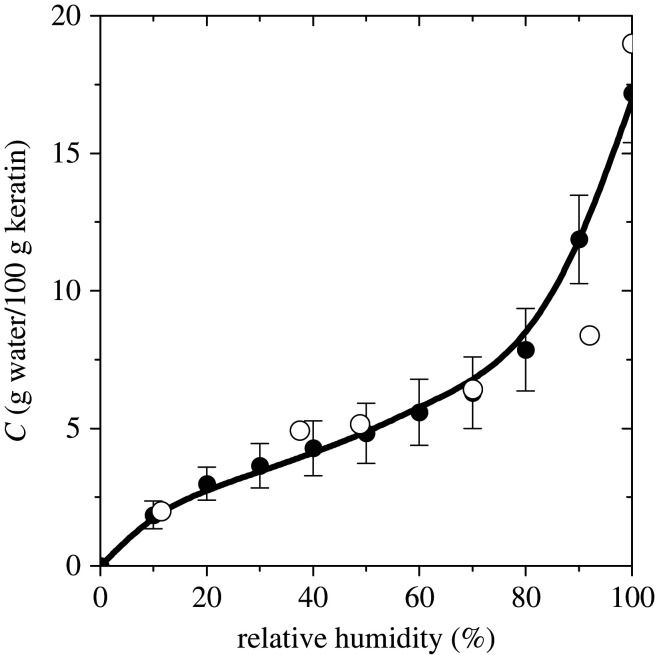
Calculated solubility of water in amorphous gecko keratin at 300 K as a function of relative humidity. The error bars have been calculated as the mean-square deviation in the number of water molecules (per 100 g keratin) about its average. The filled markers are the results of the GCE simulations and the curve is a fit to the GAB model [28] with the following coefficients: *C*_m_ = 3.20 g water per 100 g keratin, *K* = 13.51 and *b* = 0.81. The open symbols are experimental data for the dorsal and ventral scales of the snake *Naja nigricollis*, which as for any reptilian integument are made of β-keratin.

We started from water–keratin mixtures of specified concentration, initially relaxed in the *NPT* ensemble for 50 ns (for the details of generation of relaxed keratin samples, the reader is referred to [23]). At a fixed chemical potential of water, we did a GCE simulation of the gecko keratin phase. Pressure-expanding the chemical potential of water in the gecko keratin phase, equation (3.2), we did a second GCE simulation of the pure water vapour phase, to find the phase coexistence point (i.e. the water vapour density, or RH, at the same value of the chemical potential). Because both phases have the same temperature, the temperature-dependent term $k_{B}T\ln[{\left(h^{2}/2\pi mk_{B}T\right)}^{3/2}]+B(T)$ in equation (2.4) in both phases cancels. In order to reduce errors associated with the expansion of the chemical potentials in terms of pressure, equation (3.2), at different RHs we did a few test simulations in the GCE to find water concentrations close to the equilibrium concentrations. Then we did GCE simulations on water–keratin mixtures, with water contents near the equilibrium value for 30 ns to find water concentrations at each RH, consistent with the fixed values of *μ*, *V* and *T*. The calculated sorption isotherm of water in the gecko keratin at 300 K is shown in figure 1. During the course of GCE simulation, water molecules are exchanged between an ideal gas reservoir of water molecules and the system (water–keratin mixture). At equilibrium the density fluctuated around an average value. The error bars in figure 1 are calculated from density fluctuations around the average.

We have further summarized, in table 1, the density and the volume percent of water as well as the number of water molecules per amino acid in the keratin phase, as a function of RH. The sorption isotherm of water in the gecko keratin at 300 K has a general sigmoidal shape, known for water solubility in α-keratin [25,29]. The sigmoidal shape of the calculated sorption isotherm can be fitted by the GAB model [28], assuming ‘multilayer’ adsorption. In other words, water sorption in gecko keratin corresponds to an absorption behaviour that looks like the water absorbs in layers.

**Table 1.** Conversion between the solubility of water, the density of water, number of water molecules per amino acid and the volume percent of water in the keratin phase and the relative humidity^a^.

RH (%)	solubility (g water/100 g keratin)	number density of water (nm^−3^)	number of water molecules per amino acid	volume percent of water (%)
10.0	1.85	0.758	0.099	0.97
20.0	2.99	1.215	0.159	1.85
30.0	3.64	1.477	0.194	2.06
40.0	4.28	1.733	0.229	2.54
50.0	4.82	1.951	0.258	2.62
60.0	5.59	2.242	0.298	3.28
70.0	6.29	2.511	0.336	3.83
80.0	7.86	3.098	0.420	5.08
90.0	11.87	4.519	0.634	8.33
100.0	17.19	6.237	0.918	12.65

^a^The vapour pressure of water at 300 K is 3.55 kPa [40,46].

Experimental or computational reports on the water solubility in the gecko β-keratin, with which to compare our calculations, are scarce. We have experimentally measured the water uptake of dorsal and ventral scales of the snake *Naja nigricollis* and the claw tips of the Tokay gecko *Gekko gecko* toes (≈ 30 g water per 100 g sample at 100% RH) at 297 K and 100% RH. The calculated solubilities are compared with experimental data for scales of the snake *Naja nigricollis* in figure 1. The agreement of calculated values with experiment is rather good. On the other hand, one should note that even a small error of the order of ≈ 0.7*k*_B_*T* in the calculation of chemical potential leads to deviations of calculated solubilities by a factor of 2.

The water solubilities found are also in line with those in other structural proteins: the silk of the silkworm *Bombyx mori* absorbs 12 and 20 (g water per 100 g silk) in its amorphous [47] and β-sheet [48] regions at 75% RH, respectively. Compared to the α-keratin (which uptakes ≈ 30 wt% water at 300 K and 100% RH), a lower water solubility for the amorphous keratin sample, examined in this work, is expected.

### 4.2. Swelling of the gecko keratin

Water sorption in the gecko keratin causes its swelling. The swelling ratio is defined as

**Figure 2.**
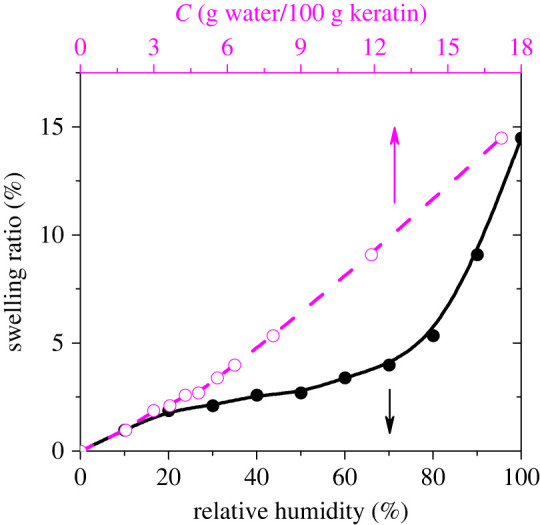
Dependence of the swelling ratio of the gecko keratin on the relative humidity and on the water content of keratin.

4.1 $$\mathrm{Swelling}\;\mathrm{ratio}=\;\left(\frac{V_{\mathrm{mixture}}-V_{\mathrm{keratin}}}{V_{\mathrm{keratin}}}\right)\;\times 100,$$

where *V*_mixture_ is the volume of keratin–water mixture and *V*_keratin_ is the volume of dry keratin. We show in figure 2 the swelling ratio of gecko keratin as a function of the RH and the water content. The swelling ratio versus the RH has a sigmoidal shape, in accordance with the sorption isotherm. It depends, however, linearly on the water content of keratin. As a result of water sorption, the distance between amino acid moieties increases. This leads to the weakening of the hydrogen bond network in keratin. A further increase in the RH causes a further decrease of the keratin density and more disruption of its hydrogen bond network.

### 4.3. Mechanical properties of the gecko keratin

The water content of keratin has been discussed as an important factor affecting the mechanism of increased wet adhesion in gecko keratin. The elastic properties of the keratin describe its response to external stress in the elastic limit, and hence insight into the structural stability under deformation. Despite the importance of the elastic properties of the gecko keratin in a variety of contexts including wet adhesion, experimental reports on them as a function of RH are scarce. To address this deficiency, here we establish a connection between elastic properties of the gecko keratin and the RH. We calculate Young's modulus and Poisson's ratio as a function of RH to clarify whether the variation of such elastic properties with the RH could be responsible for the increased adhesion of the gecko keratin at higher humidity conditions.

To calculate Young's modulus, a uniaxial deformation was applied along one of the axes; the length of the simulation box along the deformation direction was stretched at a constant rate of 0.00001 nm ps^−1^ = 1.0 cm s^−1^ and the coordinates of all atoms were rescaled accordingly. The system is coupled anisotropically to a Berendsen barostat [35]; along the deformation axis, incompressibility is imposed, and along the other two axes the isothermal compressibility was set to 10^−6^ kPa^−1^. This means that the dimensions of the simulation box perpendicular to the stretching direction were allowed to adjust as a result of coupling to the pressure, to ensure that the orthogonal directions did not contribute to the stress–strain behaviour. The strain is defined as

**Figure 3.**
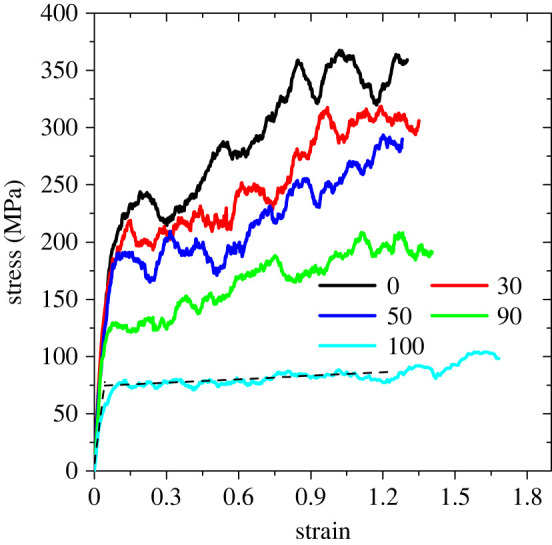
Stress–strain relationship for the gecko keratin at relative humidities shown in the legend. The intersection of the dashed lines (tangents to the elastic and strain hardening regions) shows the yield point.

4.2 $$\varepsilon =\frac{L_{∥}(t)-L_{∥}(0)}{L_{∥}(0)},$$

where $L_{∥}(t)$ and $L_{∥}(0)$ are the dimensions of the simulation box in the stretching direction at time *t* and at zero deformation (*t* = 0), respectively. During the stretch test, the components of the pressure tensor and the dimensions of the simulation cell were recorded every 0.75 ps. The response component of the stress tensor along the deformation direction, as a result of imposed strain, is shown in figure 3.

The stress–strain relationship at low RHs resembles that of deformed polymer glasses. An initial elastic response is followed by yielding, strain softening and strain hardening. At the same strain, the stress decreases with increasing RH. This is in agreement with previous experimental findings on gecko setae [11] and silk [48]. At high RHs (100%), the stress response resembles that of rubber, without a definite yield point. At low strains, less than 0.02, an elastic regime is seen, over which stress varies linearly with strain. We have calculated the slope (Young's modulus) over this linear regime (table 2). The results show that the dry gecko keratin is stiffer than the wet samples; the softness increases with increasing RH. A marked decrease in Young's modulus (i.e. an increase in softness) is observed at RH greater than 80%. Our calculated values of Young's moduli are close to the available experimental values; Young's moduli of gecko seta at 30% and 80% RHs are 3.7 GPa and 2.13 GPa [11], both in quite good agreement with our calculations, 4.2 GPa and 3.5 GPa, respectively.

**Table 2.** Mechanical properties of gecko keratin as a function of the relative humidity, at 300 K.

RH (%)	Young’s modulus (GPa)	yield		failure		Poisson's ratio
		stress (MPa)	strain	stress (MPa)	strain	
0.0	4.65	241	0.195	345	0.997	0.405
10.0	4.45	229	0.177	318	1.042	0.412
20.0	4.32	222	0.162	302	1.086	0.419
30.0	4.20	217	0.149	286	1.135	0.425
40.0	4.07	198	0.137	273	1.148	0.428
50.0	3.92	191	0.123	263	1.159	0.432
60.0	3.78	174	0.114	243	1.196	0.436
70.0	3.67	154	0.102	231	1.229	0.439
80.0	3.50	142	0.091	215	1.302	0.445
90.0	2.97	128	0.079	182	1.407	0.468
100.0	2.10	75	0.071	95	1.594	0.498

We have also calculated the yield points (table 2). At high RHs, the yield point is defined as the intersection of the two tangents to the elastic and strain hardening regions (figure 3 at 100% RH). In agreement with experiment [11], the yield stress decreases and the yield strain shifts to lower values with increasing RH. These findings confirm the softening effect of water on gecko keratin.

**Figure 4.**
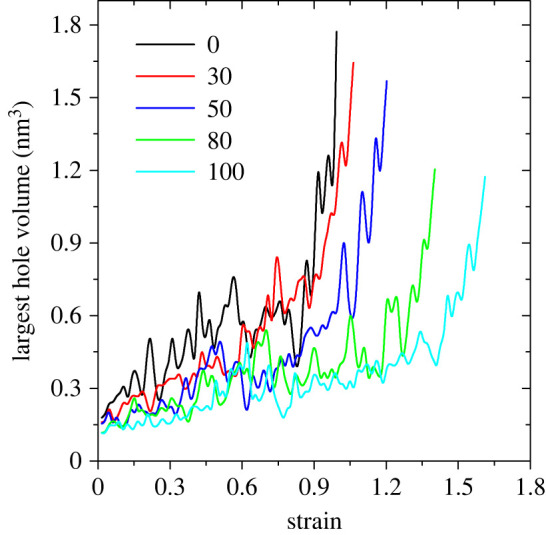
The volume of the largest void as a function of strain, at relative humidities shown in the legend. The curves for different relative humidities are plotted up to a strain at which the volume of the largest void grows to ten times of that of the unstrained sample.

At high deformations, cavities are formed which upon further deformation cause crack formation and eventually material failure. Because the classical force field employed in our simulations does not allow bond breaking or formation, we cannot directly calculate the stress/strain at failure. We define the fracture point as a state, where the volume of the largest void is 10 times larger than that of the initially unstrained system, to characterize failure stress/strain as a function of RH (table 2). The strain at failure grows from ≈ 1.0 for dry keratin to ≈ 1.6 for wet keratin at 100% RH. Also, the stress at failure decreases from 345 MPa for dry keratin to 95 MPa for wet keratin at 100% RH. Although the values of stress/strain at failure are not comparable to experiment [11] in absolute terms, the trend of their decrease/increase with the RH is in agreement with experiment. We also show the strain dependence of the volume of the largest void in the gecko keratin in figure 4. The curves in figure 4 are plotted up to the strain at which the volume of the largest void becomes ten times of that of the unstrained sample. The results show that at the same strain, the volume of the largest void is smaller at higher RHs. A smoother increase in the volume of the largest void with the strain is observed at higher RHs. Also, fluctuations in the volume of the largest void decrease with increasing RH.

**Figure 5.**
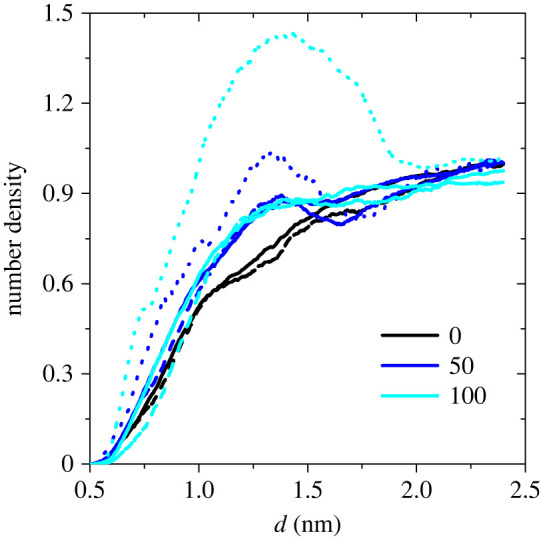
Number density (normalized with the corresponding bulk value) profiles for all keratin atoms (full curves), N and O atoms of keratin (dashed curves), and O atom of water (dotted curves) as a function of distance from the centre of the cavity, *d*. The relative humidities are shown in the legend.

To further elucidate the softening effect of water on keratin, we show in figure 5 the density profiles (normalized with the corresponding bulk values) for all keratin atoms, for O and N atoms of keratin, and for water as a function of distance from the centre of the cavity in strained dry and wet (0%, 50% and 100% RH) keratin samples. The average cavity radius in all three cases is 0.65 nm. Compared to the density profiles for all atoms, those for keratin N and O atoms are slightly shifted to larger distances; i.e. the nonpolar atoms of the amino acid side chains preferentially locate at the keratin–vacuum interface. In wet keratin, water molecules connected to the polar keratin groups occupy the cavity surface. At the interface, the dry keratin has a lower density than the wet keratin. This means that compared to wet keratin, the dry keratin becomes thinner and, therefore, fails more easily at the cavity–keratin interface. The density profile peak for water starts at shorter distances than that for keratin and it has a maximum at the keratin–cavity interface. Water occupies the inner surface of the cavity and hydrates the keratin at the interface. This effect is more pronounced at higher RHs where water molecules contact the hydrophilic groups of keratin. This has a stabilizing effect on the strained wet keratin, describing the increased strain at failure with increasing RH.

We have also calculated Poisson's ratio, *ν*, which is defined as

4.3 $$\nu =-\frac{\left(L_{⊥}(t)-L_{⊥}(0))/L_{⊥}(0\right)}{\left(L_{∥}(t)-L_{∥}(0))/L_{∥}(0\right)},$$

where *L*_⊥_ is the length of simulation box in the directions perpendicular to stretching, *L*_⊥_ = *A*^1/2^, *A* being the area perpendicular to stretching. The calculated values of Poisson's ratio increase with increasing RH from that typical of an elastomer (0.4) to that of an incompressible liquid (0.5).

**Figure 6.**
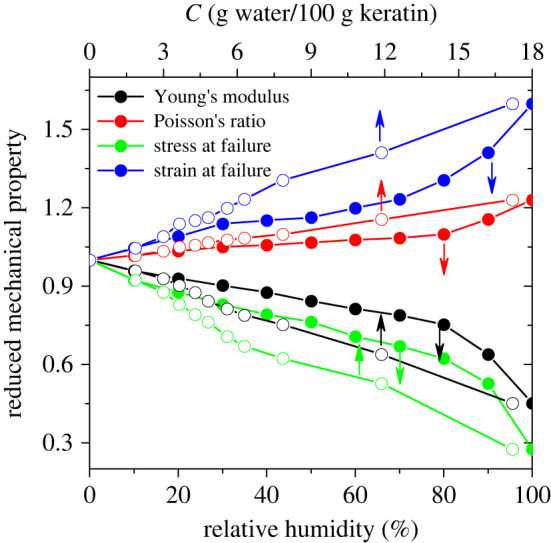
Dependence of Young's modulus, Poisson's ratio, stress at failure and strain at failure of gecko keratin on the relative humidity (filled markers) and on the water content of keratin (open markers). All mechanical properties are reduced in terms of the corresponding values for pure keratin, reported in table 2.

We show in figure 6 the dependence of mechanical properties on the RH and on the water content of keratin. While a marked change in the mechanical properties occurs at RHs > 80%, a nearly linear relationship between them and the water content of keratin is observed. A comparison of the influence of water on the Young's modulus and the Poisson ratio shows that while the Young's modulus of gecko keratin decreases by a factor of 2.2 at 100% RH, its Poisson ratio increases by about 20%. In other words, the effect of RH (water content) on the Young's modulus is more pronounced than on the Poisson ratio.

Our calculated mechanical properties reveal that the water has a softening effect on the gecko keratin. At low RHs (less than 80%), the plasticization by water is not pronounced. At high RHs, the water molecules penetrated between the amino acid chains cause more swelling of keratin and decrease the intermolecular interaction between protein chains. This has a weakening effect on amino acid interactions and softens the keratin.

## 5. Conclusion

We have performed GCE molecular dynamics simulations to calculate the water content of gecko keratin as a function of RH. The calculated sorption isotherm of water in the gecko keratin at 300 K has a sigmoidal shape, similar to that of water solubility in α-keratin [25,29]. We have also experimentally measured water uptake of dorsal and ventral scales of the snake *Naja nigricollis*. The calculated water uptake of the gecko keratin is in good agreement with experiment. The calculated water solubilities in amorphous gecko keratin are also consistent with those in α-keratin, for which experimental data exist in the literature [25,29].

To examine the softening effect of water on the gecko keratin, we have calculated its mechanical properties as a function of RH. Young's modulus of gecko keratin decreases with increasing RH. Below 80% RH, the decrease in Young's modulus is small, but picks up substantially above 80% (by a factor of 2.2 at 100% RH, compared to that for the dry keratin). The yield stress and strain both decrease with increasing RH. Increasing the RH decreases the sizes of voids that appear in the sample upon straining. Using the relative increase in the size of voids in the strained, compared to the unstrained, keratin as a surrogate for material failure, we noticed that increasing RH causes an increase in the strain and a decrease in the stress at failure. All findings are in agreement with experiment [11]. The change in the mechanical properties is only pronounced at high RHs (greater than 80%). However, a nearly linear relationship between mechanical properties and the water content of gecko keratin was found.

The sorbed water in keratin causes swelling of keratin and decreases the direct contact between amino acids of different peptide chains. High RHs cause more swelling of the keratin and decrease the intermolecular interaction between amino acid chains. This has a weakening effect on amino acid interactions and leads to the keratin softening.

## Data Availability

This article has no additional data.
